# Morphology, Mechanical Properties and Dimensional Stability of Biomass Particles/High Density Polyethylene Composites: Effect of Species and Composition

**DOI:** 10.3390/polym10030308

**Published:** 2018-03-13

**Authors:** Binshan Mu, Haigang Wang, Xiaolong Hao, Qingwen Wang

**Affiliations:** 1 Key Laboratory of Bio-based Material Science and Technology (Ministry of Education), College of Material Science and Engineering, Northeast Forestry University, 26 Hexing Road, Harbin 150040, China; mubinshan@163.com (B.M.); haoxiaolong.nefu@hotmail.com (X.H.); 2 College of Materials and Energy, South China Agricultural University, 483 Wushan Road, Guangzhou 510642, China

**Keywords:** biomass species, chemical component, mechanical properties, water absorption, dynamic mechanical properties

## Abstract

The utilization of four types of biomass particles, including hardwood (poplar), softwood (radiata pine), crop (wheat straw) and bamboo (moso bamboo), as reinforcing fillers in preparing high density polyethylene (HDPE) based composites was studied. To improve interfacial compatibility, maleic anhydride grafted polyethylene (MAPE) was applied as the coupling agent. The effects of the biomass species on the mechanical and water absorption properties of the resulting composites were evaluated based on chemical composition analysis. A creep-recovery test was conducted in single cantilever mode using a dynamic mechanical analyzer. Results show that the four types of biomass particles had similar chemical compositions but different composition contents. Poplar particles with high cellulose content loading in the HDPE matrix exhibited higher tensile and flexure properties and creep resistance. Fracture morphology analysis indicated a weak particle-matrix interface in wheat straw based composites. Given the high crystallinity and minimum hemicellulose content, the moso bamboo reinforced composite showed high impact strength and better water resistance.

## 1. Introduction

Wood plastic composites (WPCs) have been widely used in automotive components, furniture and packaging given their environmentally-friendly features. Wood fibers, as WPC reinforcers, have many advantages over glass and other synthetic fibers in low cost, renewability, availability and biodegradability [1]. With continually expanding applications, diverse biomass materials—along with wood residues—are used for preparing WPCs such as crop, hemp, cotton, jute, sisal and husk [2].

The properties of WPCs, with an identical polymer matrix and processing conditions, depend mostly on the physical and mechanical characteristics of the particles and the chemical interaction between particles and polymers [3]. To improve the compatibility between hydrophilic cellulosic materials and hydrophobic polymers, coupling agents such as maleic anhydride grafted polyethylene and maleic anhydride grafted polypropylene are usually applied during composite processing [4]. Strong compatibility contributes to the dispersion and sufficient wetting of particles in matrices to form strong interface adhesion. The improved fiber-matrix interaction due to ester linkages facilitates the transfer of stress from the matrix to the reinforcing particles, thus resulting in improved mechanical properties. Moreover, sufficient interface adhesion reduces the water absorption of WPCs [5].

Biomass material is a generic term for natural and complex polymeric composites, which generally contain cellulose, hemicellulose, lignin and extractives [6]. The unique chemical components play different roles in lignocellulose materials, such that biomass particles exhibit different mechanical and surface chemical properties, thereby affecting WPC characteristics [7]. As cellulose is the main component providing fibers with strength and structural stability, WPCs reinforced by biomass fibers with high cellulose content exhibited high elasticity modulus [8]. The chemical compositions of barley husk and coconut shell were determined by Andrzej et al. [9], they found that structural materials (cellulose, starch) content in barley husk was higher than in coconut shells and the tensile strength of composite reinforced by the former was higher than the latter. Andrzej et al. [10] also reported that wheat husk contained more surface silicon than softwood and the wheat husk composites showed 15% better Charpy impact strength than softwood composites. Ou et al. [11] demonstrated the effect of removing wood cell wall composition on the morphology, mechanical properties and dimensional stability of WPCs. They found that removing hemicellulose and lignin can reduce water absorption considerably while improving tensile strength. Alireza [12] used oak and pinewood (hot-water extracted and un-extracted) as reinforcement to prepare WPCs. Results showed that the tensile, flexural and impact properties of the resulting composite improved after removing hot water extractives. They also concluded that extractives were the main reason for low interaction between lignocellulosic materials and the coupling agent. Recently, extensive research [13,14] has been conducted to investigate the effects of individual chemical components on WPCs properties; however, natural fibers are biologically diverse and different chemical components have a combined effect on the characteristic properties of biomass materials and the chemical interaction between particles and matrices. Moreover, many biomass resources have not been well used. It is therefore necessary to identify more suitable biomass materials to prepare WPCs.

In this study, poplar, radiata pine, wheat straw and moso bamboo—which represent hard wood, soft wood, crop straw and bamboo respectively—were chemically characterized, and the use of four typical biomass particles in polyethylene was studied. The effects of four typical biomass species on bio-composites properties were investigated from a chemical component perspective. Fourier Transform Infrared (FT-IR) and X-Ray Diffraction XRD were used to study the chemical components of each species. The interfacial adhesion of composites was characterized using dynamic mechanical analysis, creep analysis and scanning electron microscopy. The mechanical properties, water uptake and dimensional swelling of the composites were determined as well.

## 2. Materials and Methods

### 2.1. Materials

Poplar and radiata pine were obtained from Heilongjiang, China and New Zealand, respectively. Wheat straw was harvested from a farm located in Harbin, Heilongjiang. Moso bamboo (with the epidermis removed) was purchased from Zhejiang, China. Different types of biomass were selected to represent hardwood, softwood, crop straw and bamboo, respectively. Biomass materials of each species (poplar and radiata pine with bark removed) were cutter-milled to the point where the product could pass through a 40 mesh sieve. HDPE (5000s) was obtained from Petrifaction Company (Daqing, China) with a density of 0.95 g/cm^3^ and a melt flow rate of 0.90 g/10 min. Maleic anhydride grafted high-density polyethylene (MAPE), obtained from Sunny (Shanghai, China) with a maleic anhydride grafting ratio of approximately 1% and a melt flow rate of 4.85 g/10 min was used as the coupling agent. Stearic acid (#1801) and polyethylene wax (Nanjing Adisi Import & Export Co., Ltd., Nanjing, China) were used as mixing lubricants at a weight ratio of 1:1.

### 2.2. Preparation of Composite Specimens

The biomass particles were dried in an oven at 103 °C for 24 h. HDPE, dry biomass particles, MAPE and lubricant were then premixed at a 60:35:3:2 ratios in a high-speed blender at room temperature for 10 min. Then the mixture was granulated using a co-rotating twin-screw extruder (SJSH30; Nanjing Rubber-Plastic Machine Ltd., Nanjing, China) at temperatures of 145–150–160–165–165–155–150 °C (from the feeder zone to the die). The resulting granules were then extruded by a single-screw extruder (SJ45; Nanjing Rubber-Plastic Machine Ltd., Nanjing, China) at temperatures of 145 °C in the melting zone, 150–160 °C in the pumping zone and 165 °C in the die zone to produce bio-composite sheets. The rotation speeds of the twin-screw and single-screw extruder were 40 and 10 rpm, respectively.

### 2.3. Characterization of Biomass Particles

#### 2.3.1. Chemical Composition

The polysaccharide, cellulose, lignin, ash and hot-water-extracted contents of different biomass particles were measured. To remove soluble extractives, oven-dried particles were bundled with filter paper and extracted for 6 h using a soxhlet extractor. The bundles were submerged in an ethanol solution and the processed particles were then treated in the solution to remove lignin and holocellulose, respectively.

The polysaccharide content was determined as the amount of residue after removing lignin from processed particles. In this procedure, NaClO_2_ and CH_3_COOH were added once per hour at 75 °C, repeated 4 times to remove lignin [15]. The cellulose content was determined by further treatment of the previously processed polysaccharide with a 17.5% NaOH aqueous solution for 40 min to remove hemicelluloses, based on TAPPI 203. The mixture was then filtered and the acetic acid aqueous solution was added in residues and kept for 20 min, after which the residue was rinsed with boiling water and filtered again. Finally, the residue was dried and weighed. Extracted particles were further treated with 15 mL of aqueous 72% sulfuric acid solution at 20 °C for 2 h; then, the distilled water was added to the mixture and boiled continuously for 4 h. The residue was then filtered, washed, dried and weighed. The final value was considered the lignin mass according to TAPPI 222.

The hot-water-extracted content was measured by placing the particles in boiling water for 3 h. The difference in quality before and after processing was considered to be the hot-water-extracted content according to TAPPI 207. Ash content was tested based on TAPPI 211 using high-temperature calcination. The crucibles carrying wood particles were put into a muffle and the temperature was increased from 25 to 600 °C at a heating rate of 20 °C/min. The particles were maintained in thermal insulation for 3 h at 600 °C and then cooled to room temperature.

#### 2.3.2. FT-IR Analysis

A Fourier transform infrared spectroscope (Nicolet 6700 FT-IR; Thermo Fisher Scientific, Agawam, MA, USA) was used to analyze the different compositions of wood flour and monosaccharides at a resolution of 4 cm^−1^ with 32 scans.

#### 2.3.3. X-ray Diffraction

X-ray diffraction (D/MAX-2200; Rigaku, Tokyo, Japan) was performed on the samples using Cu Kα radiation (λ = 0.1541 nm) at a generator voltage of 40 kV and a current of 30 mA. The scanning range was 5° to 40° with a scanning speed of 5°/min.

#### 2.3.4. Morphological Analysis

The particle geometry after processing was observed using a high-definition digital microscope (GE-5; Shanghai Changfang Optical Instrument Co., Ltd, Shanghai, China) at a smooth section along the direction of extrusion. The images were analyzed with the Nano Measure 1.2. software.

The samples were frozen in liquid nitrogen and then quickly impact-fractured. The fractured surfaces were sputtered with a thin layer of gold and observed using a scanning electron microscope (Quanta 200; FEI Co., Hillsboro, OR, USA) operated at an acceleration voltage of 12.5 kV.

#### 2.3.5. Mechanical Test

Dumbbell-shaped specimens measuring 165 mm × 13 mm × 4 mm were used to test tensile strength according to ASTM D638 and specimens measuring 80 mm × 13 mm × 4 mm were used to test flexural strength according to ASTM D790. Tensile and flexural properties were tested using a universal electromechanical testing machine (CMT5504; MTS Systems (China) Co., Ltd, Shenzhen, China). Samples measuring 80 mm × 10 mm × 4 mm were used to test impact strength in the un-notched mode according to ASTM D6110 using an impact tester (JC-5; Chengde Jingmi Testing Machine Co., Ltd, Chengde, China). For each formulation, 10 replicates were tested.

#### 2.3.6. Water Absorption

Samples measuring 20 mm × 20 mm × 4 mm were soaked in water at 25 °C for 55 days to test water uptake, according to ASTM D570. All samples were oven-dried and weighed before being immersed in water. Five replicates were used for each formulation. The water absorption (WA) and swelling in thickness (ST) of each specimen were determined at specific times using the following equations:

(1) $$WA=\frac{W_{t}-W_{0}}{W_{0}}\times 100\%$$

(2) $$ST=\frac{T_{t}-T_{0}}{T_{0}}\times 100\%$$

where *W_t_* and *W*_0_ denote the sample weight at a specific time and the initial time, respectively and *T_t_* and *T*_0_ denote the sample thickness at a specific time and the initial time, respectively.

#### 2.3.7. Creep Analysis

Samples measuring 35 mm × 12 mm × 4 mm were analyzed in a single cantilever mode at 30 and 60 °C, respectively, using a dynamic mechanical analyzer (Q800; TA Instruments Inc., New Castle, DE, USA). Creep tests were conducted by applying a 2 MPa load on the samples for 30 min to observe creep behavior. Then, the load was released for 30 min to measure the recovery behavior.

#### 2.3.8. Dynamic Mechanical Analysis (DMA)

Dynamic mechanical properties of samples measuring 35 mm × 12 mm × 4 mm were measured using a dynamic mechanical analyzer (Q800; TA Instruments Inc., New Castle, DE, USA). Tests were performed in a single-cantilever mode with an amplitude of 50 μm and frequency of 1 Hz. The temperature increased from −30 to 130 °C at a heating rate of 3 °C/min.

## 3. Results and Discussion

### 3.1. Characterization of Wood Particles

#### 3.1.1. Chemical Composition Content

The chemical composition of the investigated biomass materials was determined (Table 1). Among the four particles, poplar exhibited the highest polysaccharide and cellulose content. Lignin content was highest in radiata pine. Wheat straw demonstrated the highest hemicellulose content, hot-water extractives and residual ash but cellulose and lignin content in wheat straw was the lowest of all materials. Compared with other chemical components, the hemicellulose in moso bamboo was the lowest.

#### 3.1.2. FT-IR Analysis

**Figure 1:**
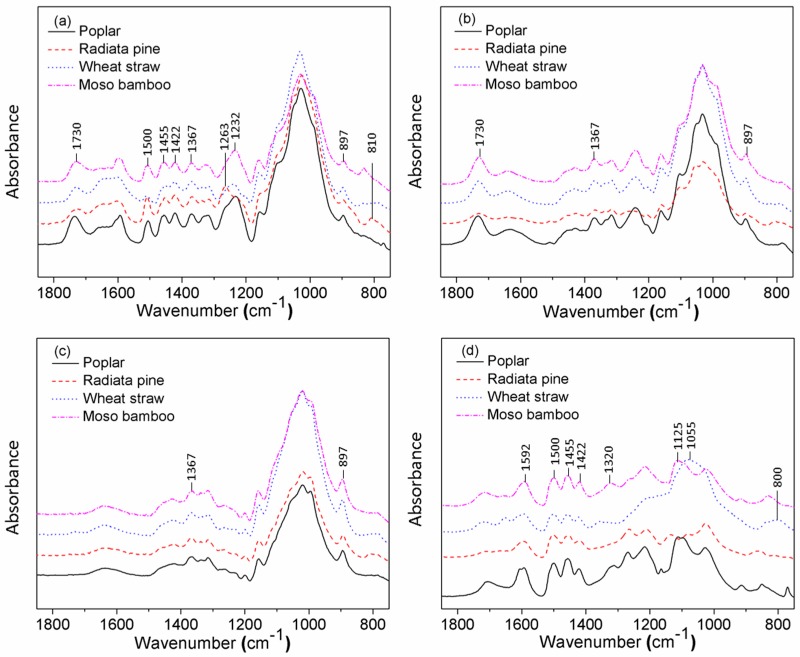
Fourier Transform Infrared (FT-IR) spectra of (**a**) biomass particles and the chemical composites extracted from each particle: (**b**) polysaccharides; (**c**) celluloses; and (**d**) lignins.

From the FT-IR spectra (Figure 1), the biomass particles showed absorption bands at 1730 cm^−1^ for unconjugated carbonyl and acetyl groups of hemicellulose [16]; 1598, 1506 and 1455 cm^−1^ for aromatic skeletal vibration from lignin; and 1370 cm^−1^ for the C–H bending vibration of cellulose and hemicellulose (Figure 1a). The cellulose present in biomass materials is thought to be common to all species, while there may be variations from one species to another in the precise nature of the lignin. Guaiacyl ring breathing with carbonyl stretching at 1263 cm^−1^ was greater in radiata pine than in other particles. The radiata pine did not show an absorption band at 1232 cm^−1^, attributed to a combination of the deformation of the syringyl nuclei and hemicellulose. The respective bands at 1320 cm^−1^ for C–O stretching from the syringyl unit and 1125 cm^−1^ for the C–H bending vibration of lignin were absent in radiata pine. These could be explained by the fact that the lignin of softwood is mainly composed of guaiacyl units; hardwood, bamboo and crop straw contain guaiacyl and syringyl units. After extracting chemical components from particles, the corresponding absorption peaks disappeared. The intensity of the band related to C_1_ group frequency vibration from cellulose and hemicellulose (893 cm^−1^) was absent from the lignin spectrum. The cellulose did not exhibit the band at 1242 cm^−1^, which belongs to the C–O stretching vibration of the acetyl group (lignin and hemicelluloses) [17]. The bands around 1055 and 800 cm^−1^ in the lignin of wheat straw are characteristic of SiO_2_ (Figure 1d), a key component of crop ash [18]. Combined with previous chemical composition content analysis (Table 1), there appeared to be little difference in the chemical components between each particle. The different component contents of each biomass type confirm lignocellulosic characteristics and species diversity.

#### 3.1.3. XRD Analysis

**Figure 2:**
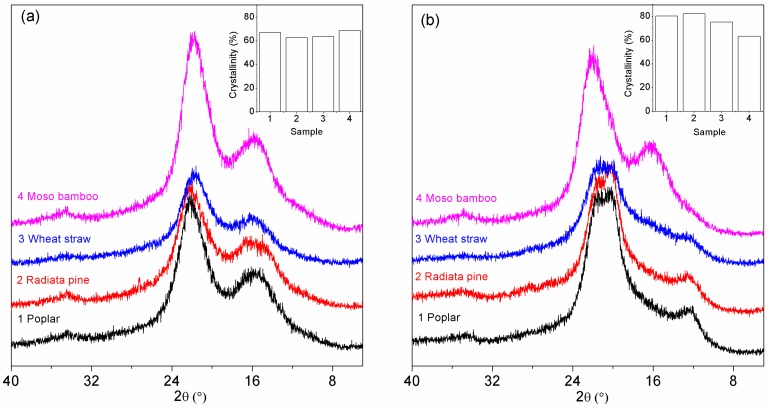
X-Ray Diffraction (XRD) patterns of (**a**) biomass particles and (**b**) celluloses that were extracted from particles.

The diffractogram of the original particles exhibited a typical crystalline structure of native cellulose I (Figure 2a). The peaks at approximately 2θ = 14°, 16°, 23° and 35° were assigned to the (101), (10$\bar{1}$), (002) and (040) crystallographic plane reflections [19], respectively. Due to large amounts of amorphous material such as lignins, hemicelluloses, pectins and amorphous cellulose, the peaks on the X-ray diffractograms at 2θ = 14° and 2θ = 16° corresponding to (101) and (10$\bar{1}$) were not observed and instead appeared as one broad peak [20]. The *CrI* of poplar, radiata pine, wheat straw and moso bamboo were 67.18%, 62.79%, 63.80% and 68.74%, respectively. The high crystallinity of moso bamboo indicated that the cellulose chains in the particles were well-ordered. Alkali treatment of the polysaccharide caused a sectional conversion of cellulose I to cellulose II as indicated by peaks at 2θ = 12.4°, 20.3° and 21.4° resulting from the (101), (10$\bar{1}$) and (002) lattice planes of cellulose II [21,22], except for moso bamboo (Figure 2b). This result presumably occurred due to moso bamboo particles with high crystallinity against liquor penetration into the cell wall, resulting in a decrease in crystallinity instead of crystal transformation. The *CrI* of the celluloses—which were extracted from each particle—were 80.20%, 82.17%, 75.31% and 63.32%. An increase in crystallinity followed from the removal of amorphous compositions [23,24]. After NaOH treatment, the hydrogen of microfibril on the crystallization surface and non-crystalline region was exposed, which may produce many hydrogen bonds and kept the amorphous region close to the crystalline region and orientation, resulting in an increase in the width of the cellulose microfibril crystallization of the cell walls [21,25]. The greater the change in crystallinity after treatment, the more amorphous material existed in the particles.

### 3.2. Characterization of Bio-Composites

#### 3.2.1. Morphological Analysis

Table 2 represents the particle sizes and shapes after extrusion. Radiata pine exhibited a large particle size and low aspect ratio. The aspect ratio of wheat straw was higher than that of the other three particles.

**Figure 3:**
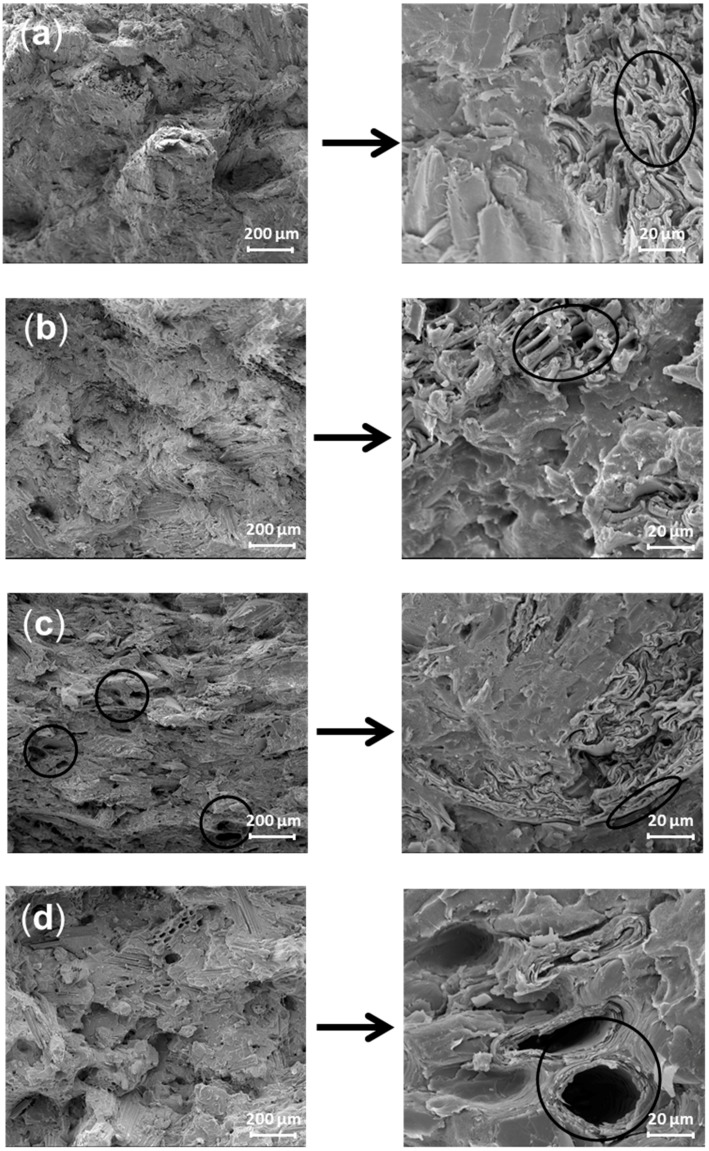
Scanning Electron Microscope (SEM) micrographs of the fractural surface of composites after creep test: (**a**) poplar/HDPE; (**b**) radiata pine/HDPE; (**c**) wheat straw/HDPE; (**d**) moso bamboo/HDPE.

After being ground, the poplar, radiata pine and moso bamboo fibers were torn, leaving an irregular, jagged surface and many open lumens (Figure 3a,b,d). The lumens of moso bamboo were larger and exhibited a circular section; however, in wheat straw/HDPE, the lumens were collapsed and had wrinkled cell walls. This result reflected the poor cell wall strength of wheat straw due to low cellulose content. As shown in Figure 3c, wheat straw particles were pulled out from the matrix and left many voids on the fracture surface; the interface between particles and the matrix was clear. By contrast, the pulled-out moso bamboo particles were covered with the HDPE matrix and exhibited a smooth surface, besides, the interface between the bamboo particles and HDPE was indistinct. According to Gao et al. [26], these behaviors are directly related to interaction adhesion. The presence of silica, lipids and hot-water extractives in wheat straw prevented interaction between particles and the coupling agent, leading to weak interface adhesion [27,28]. Moreover, the indistinct interface suggested good interfacial compatibility, which can be attributed to the high cellulose content [29]. Compared with the HDPE reinforced by moso bamboo, other composites observed from the fracture showed a loose structure. The unevenly dispersed wheat straw particles in the matrix produced aggregation may create defects in the composites [30].

#### 3.2.2. Mechanical Properties

**Figure 4:**
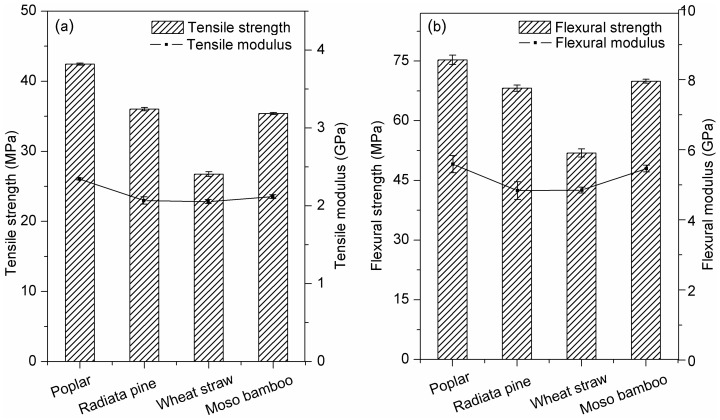
The value of the mechanical properties of composites with different species: (**a**) tensile; (**b**) flexural.

As shown in Figure 4, the highest tensile and flexural strength and modulus were exhibited in composites prepared from poplar, followed by radiata pine and moso bamboo. The wheat straw/HDPE exhibited poor tensile and flexural properties. Unlike the stress applied on neat plastic was borne by polymeric chains, biomass particles primarily bear the stress in bio-composites [26]. Thus, the excellent performance of poplar/HDPE can be attributed to high polysaccharide and cellulose contents, which contribute to particle rigidity and supply many hydroxyl groups for esterification with MAPE [7]. Moreover, the high L/D value of poplar particles may lead to an increase in strength as enhanced stress transfer. However, the wheat straw/HDPE with the highest particle aspect ratio showed poor tensile and flexural properties. It can be explained by the fact that there were fewer fiber cells that existed in the wheat straw, meanwhile, the cellulose and lignin contents were also lower than in the other three particles (Table 1). Thus, the wheat straw exhibited poor particle strength. Furthermore, the weak boundary layer, attributed to the presence of hot-water extractives, may have prevented stress transfer from the plastic matrix to the particles [31,32]. In addition, particle agglomeration is another important factor in blocking load transfer in wheat straw/HDPE, according to Ou et al. [11].

**Figure 5:**
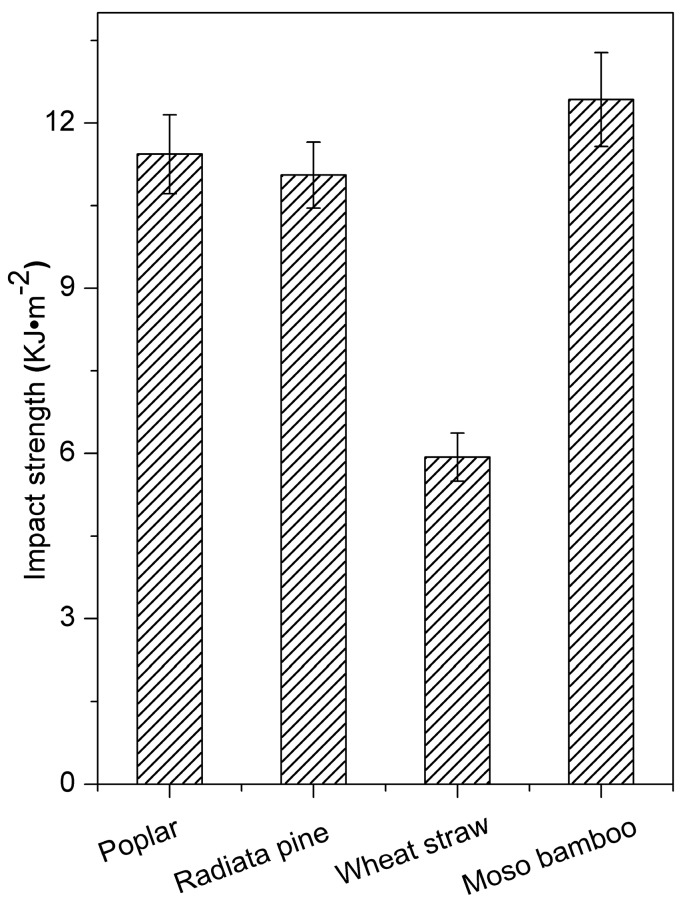
Impact strength of composites with different biomass species.

Moso bamboo/HDPE exhibited slightly lower tensile and flexural strength but higher unnotched impact strength than a similar poplar/HDPE composite (Figure 5). For unnotched impact, the total work-of-fracture can be calculated as the accumulation of reinforcing particles, the matrix and energy that dissipated due to different particle-matrix interactions (sliding, debonding, fiber pullout, etc.) [33]. The high impact strength of the bamboo-reinforced composite can be attributed to the strong interfacial bonding between the blend and biomass. In this case, the stress was more efficiently transferred from the matrices to the stiffer bamboo particles, causing an increase in energy absorption [34,35]. Beyond that, the hollow cylindrical bamboo cell (Figure 3d) played an energy-absorbing role in the polyethylene matrix, similar to a crash box. Wheat straw/HDPE exhibited the lowest impact strength compared to the other composites, possibly because the particles were not sufficiently wetted by the polymer, resulting in fiber agglomeration and blocked load transfer [36]. Moreover, the small wheat straw particle size did not lead to high impact strength because the extractives in wheat straw played a role in determining the crack initiation process by lowering the interaction between particles and the coupling agent [12].

#### 3.2.3. Water Absorption

**Figure 6:**
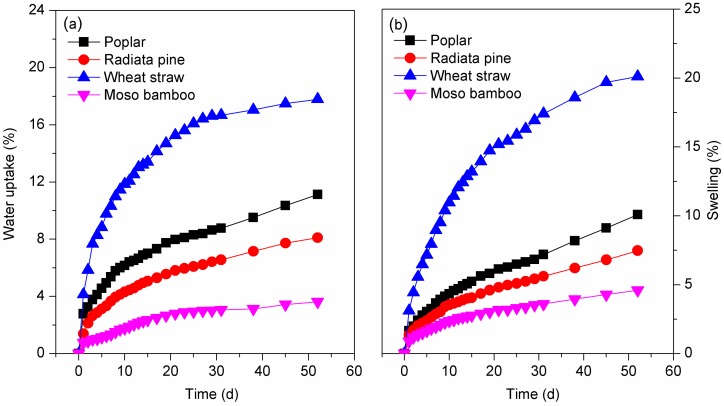
(**a**) Water absorption and (**b**) thickness swelling of the composites.

The final water absorption and thickness swelling of the composites with four biomass species decreased as follows: wheat straw/HDPE > poplar/HDPE > radiata pine/HDPE > moso bamboo/HDPE (Figure 6). The composite based on wheat straw exhibited greater degrees of water uptake and dimensional swelling, about 5 and 4.3 times higher than moso bamboo. The polyethylene matrix, which is hydrophobic, is considered the nonabsorbent part of bio-composites; therefore, the water uptake of fiber-based composites can be attributed to the availability of free hydroxyl groups on the particle surface. When immersing the composites in water, the free –OH groups in cellulose, hemicelluloses and lignin formed hydrogen bonds with water molecules [37]. In this case, hemicellulose is considered the main polymer of lignocellulosic materials responsible for water absorption due to its available hydroxyl groups [38]. Table 1 indicates that the hemicellulose content of wheat straw and poplar was higher than in radiata pine and moso bamboo, partially explaining the high water absorption in bio-composites. Due to capillarity, the small wheat straw particle size and high aspect ratio may also accelerate the penetration of water into cell lumen. The interfacial compatibility demonstrated via scanning electron microscopy (SEM) was another important factor in moisture absorption: the incompatibility between wheat straw particles and the matrix provided pathways for moisture uptake and increased the interaction between water molecules and free –OH. Different particle structures may also contribute to different degrees of water uptake, especially dimensional swelling of the composites. During absorption, water uptake occurred on the surface of the particles at first and then the water transferred from one cell to another over time. Compared with bamboo, wood cells are highly porous; the pits interlinking adjacent cells in wood cell walls may cause an increase in wood permeability and water penetration [39]. In terms of osmosis, the large vertical vessels of bamboo were closely surrounded by fiber cells and prevented penetration. Furthermore, the high *CrI* of moso bamboo particles indicated well-ordered cellulose, which decreases hygroscopic capacity.

#### 3.2.4. Creep Analysis

**Figure 7:**
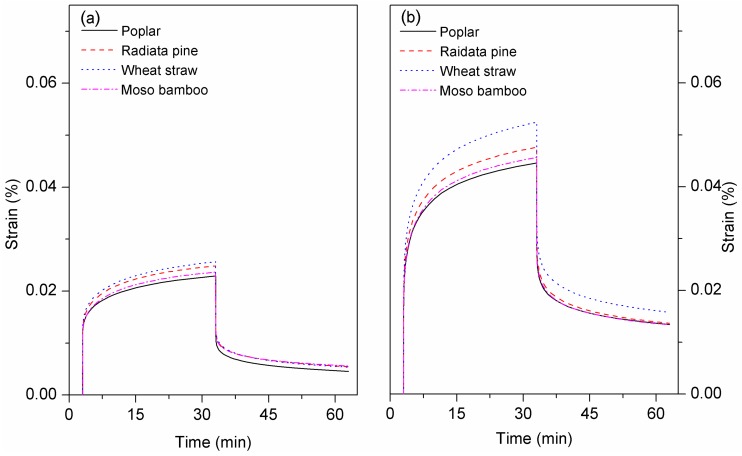
Creep-recovery curves of the composites at (**a**) 30 °C and (**b**) 60 °C.

Figure 7 displays the relative creep of the materials. The composites exhibited a decreasing order of creep deformation at 30 °C: wheat straw/HDPE > radiata pine/HDPE > moso bamboo/HDPE > poplar/HDPE. They showed a similar trend at 60 °C, consistent with the trend in mechanical strength. The creep curve can be considered a superposition of three parts: initial instantaneous deformation due to elastic deformation; viscoelastic deformation, which occurred when the deformation rate reduced gradually; and the linear section, showing viscous deformation (the slope of the line was considered the steady creep rate). During deformation, the composites with a high fiber content reflected rigidity and large elastic deformation resilience can be attributed to the biomass particles [40]. The greater deformation resistance of poplar/HDPE can be mainly ascribed to high crystallinity and cellulose content, which supplied cell walls with rigidity and provided better reinforcement. Besides, the uniform dispersion of particles in the matrix was beneficial for stress transfer and the strong interfacial bonding constrained the motion of matrix molecules in the interfacial layer, thereby reducing deformation [41,42]. The poor performance of wheat/HDPE likely resulted from the weak boundary layer, namely due to the presence of wax and hot-water extractives. Under a large load, the molecular chain of HDPE glided quickly; the combination of unencapsulated particles and the HDPE matrix was easily destroyed and resulted in unrecovered deformation. The deformation tested at 60 °C was higher than at 30 °C. This indicates that the high temperature accelerated the creep response because increased thermal energy allowed polymer chains to undergo translational motions [43].

#### 3.2.5. Dynamic Mechanical Analysis

**Figure 8:**
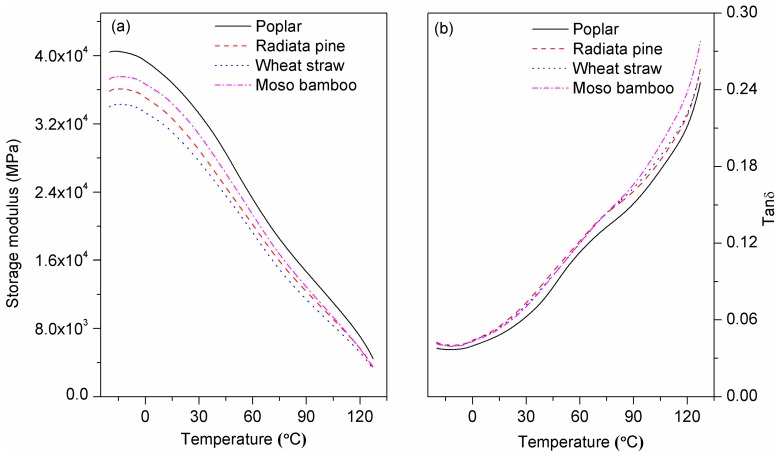
(**a**) Storage modulus and (**b**) tanδ of the composites with different biomass species.

The storage modulus (E’) of the composite filled with poplar was highest at room temperature, followed by moso bamboo/HDPE, radiata pine/HDPE and wheat straw/HDPE (Figure 8a). The result corresponded with the tensile modulus, as shown in Figure 4. The high storage modulus of poplar and moso bamboo-reinforced composites can be explained by the high cellulose content and well-ordered cellulose chains, which contributed to particle stiffness. Moreover, strong interfacial adhesion facilitated efficient stress transfer from the matrix to wood particles [44]. The E’ values decreased along with elevating temperature. Since E’ is the contribution of the elastic component of the composites and a high modulus is attributed to the stiffness created by adding wood particles into the polyethylene matrix [45]. At a low temperature, the composite stiffness depends on the density of the composites and the stiffness of matrix (crystallinity) and particles; however, as the temperature increases, particle stiffness will dominate. This is to be expected, because the mobility of the bulk matrix in composites increases at high temperatures and the difficult-to-move macromolecular cells can only store energy [46,47].

At room temperature, few differences emerged between composites in terms of the damping factor tanδ (E”/E’) amplitude (Figure 8b). Poplar/HDPE—which had a strongly bonded interface between particles and the matrix—tends to dissipate little energy, demonstrating a lower tanδ magnitude than the composites with poor interfacial adhesion [48]. Moreover, the poplar particles with high stiffness—due to high polysaccharide and cellulose contents—constrained the mobility of matrix molecules, resulting in low tanδ amplitude [49]. As the temperature increased, so did the difference. The high tanδ value of moso bamboo/HDPE at high temperatures may have been due to its excellent buffering capacity, which indicates a high degree of molecular mobility along with higher impact strength, toughness and energy dissipation.

## 4. Conclusions

The influence of biomass species and chemical components on the mechanical properties, moisture absorption, creep analysis and dynamic mechanical analysis (DMA) properties of composites were evaluated. The creep behavior and DMA of the resulting composites followed the general trend observed in static mechanical tests, in which poplar/HDPE showed better tensile and flexural properties due to the high cellulose content in poplar. The hot-water-extracted content in wheat straw particles was 2.7 and 1.7 times higher than in wood (poplar and radiata pine) and moso bamboo, respectively. The interfacial compatibility of wheat straw/HDPE was influenced by the presence of hot water extractives and the composite exhibited poor mechanical properties and water resistance. Given the high crystallinity and low hemicellulose content, moso bamboo/HDPE showed 110% better impact strength and 14.18% lower water absorption than wheat straw/HDPE. Compared to the other three composites, the bio-composites reinforced by radiata pine exhibited neither the best nor the worst properties.

## Figures and Tables

**Table 1:** Chemical properties of biomass particles used in bio-composites production.

Chemical Components	Poplar	Radiata Pine	WHEAT Straw	Moso Bamboo
Polysaccharide (%)	78.48 ± 0.30	73.79 ± 0.41	72.14 ± 0.24	71.75 ± 0.65
Hemicellulose (%)	31.73 ± 0.72	27.48 ± 0.66	32.93 ± 0.26	25.38 ± 0.65
Cellulose (%)	46.74 ± 0.89	46.31 ± 1.06	40.10 ± 0.13	46.37 ± 0.41
Lignin (%)	23.92 ± 0.18	28.84 ± 0.46	18.39 ± 0.43	26.44 ± 0.41
Hot water extracts (%)	3.89 ± 0.59	3.90 ± 0.52	10.60 ± 0.59	6.33 ± 0.40
Ash (%)	1.67 ± 0.11	2.35 ± 0.31	5.29 ± 0.28	0.74 ± 0.14

**Table 2:** Particle sizes and shapes after processing.

Species	Particle Size (μm)	Aspect Ratio ^1^ (L/D)
Poplar	122.96 ± 39.65	5.82 ± 2.50
Radiata pine	182.47 ± 51.66	4.10 ± 1.58
Wheat straw	93.54 ± 27.07	6.59 ± 2.02
Moso bamboo	133.60 ± 34.32	4.32 ± 1.56

^1^ The ratio of particle length to its diameter (L = mean length; D = mean diameter).

## References

[1] Wang S., Lu A., Zhang L. (2016). Recent advances in regenerated cellulose materials. Prog. Polym. Sci..

[2] Yao F., Wu Q., Lei Y., Xu Y. (2008). Rice straw fiber-reinforced high-density polyethylene composite: Effect of fiber type and loading. Ind. Crops Prod..

[3] Kim J.W., Harper D.P., Taylor A.M. (2009). Effect of wood species on the mechanical and thermal properties of wood-plastic composites. J. Appl. Polym. Sci..

[4] Hosseinaei O., Wang S.Q., Taylor A.M., Kim J.W. (2012). Effect of hemicellulose extraction on water absorption and mold susceptibility of wood-plastic composites. Int. Biodeterior. Biodegrad..

[5] Mohanty S., Nayak S.K., Verma S.K., Tripathy S.S. (2004). Effect of MAPP as a coupling agent on the performance of jute-PP composites. J. Reinf. Plast. Compos..

[6] John M.J., Thomas S. (2008). Biofibres and biocomposites. Carbohydr. Polym..

[7] Hassine B., Ahmed K., Patrick P., Alain C., Bernard R. (2008). Analysis of among-species variability in wood fiber surface using DRIFTS and XPS: Effects on esterification efficiency. J. Wood Chem. Technol..

[8] Shebani A.N., Van Reenen A.J., Meincken M. (2009). The effect of wood species on the mechanical and thermal properties of wood–LLDPE composites. J. Compos. Mater..

[9] Bledzki A.K., Mamun A.A., Volk J. (2010). Barley husk and coconut shell reinforced polypropylene composites: The effect of fibre physical, chemical and surface properties. Compos. Sci. Technol..

[10] Bledzki A.K., Mamun A.A., Volk J. (2010). Physical, chemical and surface properties of wheat husk, rye husk and soft wood and their polypropylene composites. Compos. Part A.

[11] Ou R., Xie Y., Wolcott M.P., Sui S., Wang Q. (2014). Morphology, mechanical properties and dimensional stability of wood particle/high density polyethylene composites: Effect of removal of wood cell wall composition. Mater. Des..

[12] Ashori A., Nourbakhsh A. (2010). Reinforced polypropylene composites: Effects of chemical compositions and particle size. Bioresour. Technol..

[13] Ou R.X., Xie Y.J., Wang Q.W., Sui S.J., Wolcott M.P. (2014). Thermal, crystallization and dynamic rheological behavior of wood particle/HDPE composites: Effect of removal of wood cell wall composition. J. Appl. Polym. Sci..

[14] Hosseinaei O., Wang S., Rials T.G., Xing C., Zhang Y. (2011). Effects of decreasing carbohydrate content on properties of wood strands. Cellulose.

[15] Nobuta K., Teramura H., Ito H., Hongo C., Kawaguchi H., Ogino C., Kondo A., Nishino T. (2015). Characterization of cellulose nanofiber sheets from different refining processes. Cellulose.

[16] Owen N.L., Thomas D.W. (1989). Infrared studies of “hard” and “soft” woods. Appl. Spectrosc..

[17] Huang L., Mu B., Yi X., Li S., Wang Q. (2016). Sustainable use of coffee husks for reinforcing polyethylene composites. J. Polym. Environ..

[18] Yao J., Xu X.W., Feng Y.Y. (2003). FTIR studies on the chemical composition of wheat straw in different layers. Spectrosc. Spect. Anal..

[19] Oudiani A.E., Chaabouni Y., Msahli S., Sakli F. (2011). Crystal transition from cellulose I to cellulose II in NaOH treated *Agave americana* L. Fibre. Carbohydr. Polym..

[20] Perel J. (1990). An X-ray study of regain-dependent deformations in cotton crystallites. J. Text. Inst..

[21] Zhou L.M., Yeung K.W., Yuen C.W.M., Zhou X. (2004). Characterization of ramie yarn treated with sodium hydroxide and crosslinked by 1,2,3,4-butanetetracarboxylic acid. J. Appl. Polym. Sci..

[22] Jähn A., Schröder M.W., Füting M., Schenzel K., Diepenbrock W. (2002). Characterization of alkali treated flax fibres by means of FT Raman spectroscopy and environmental scanning electron microscopy. Spectrochim. Acta Part A Mol. Biomol. Spectrosc..

[23] Kaushik A., Singh M., Verma G. (2010). Green nanocomposites based on thermoplastic starch and steam exploded cellulose nanofibrils from wheat straw. Carbohydr. Polym..

[24] Yu Z., Jameel H., Chang HM., Park S. (2011). The effect of delignification of forest biomass on enzymatic hydrolysis. Bioresour. Technol..

[25] Chen Q., Soykeabkaew N., Ni X., Peijs T. (2008). The effect of fibre volume fraction and mercerization on the properties of all-cellulose composites. Carbohydr. Polym..

[26] Gao H., Xie Y., Ou R., Wang Q. (2012). Grafting effects of polypropylene/polyethylene blends with maleic anhydride on the properties of the resulting wood–plastic composites. Compos. Part A.

[27] Hornsby P.R., Hinrichsen E., Tarverdi K. (1997). Preparation and properties of polypropylene composites reinforced with wheat and flax straw fibres: Part I fibre characterization. J. Mater. Sci..

[28] Hornsby P.R., Hinrichsen E., Tarverdi K. (1997). Preparation and properties of polypropylene composites reinforced with wheat and flax straw fibres: Part II analysis of composite microstructure and mechanical properties. J. Mater. Sci..

[29] Xie Y., Xiao Z., Grüneberg T., Militz H., Hill C.A.S., Steuernagel L., Mai C. (2010). Effects of chemical modification of wood particles with glutaraldehyde and 1,3-dimethylol-4,5-dihydroxyethyleneurea on properties of the resulting polypropylene composites. Compos. Sci. Technol..

[30] Ayrilmis N., Kaymakci A. (2013). Fast growing biomass as reinforcing filler in thermoplastic composites: Paulownia elongata wood. Ind. Crop. Prod..

[31] Saputra H., Simonsen J., Li K. (2004). Effect of extractives on the flexural properties of wood/plastic composites. Instrum Sci. Technol..

[32] Yi S., Xu S., Fang Y., Wang H., Wang Q. (2017). Effect of matrix modification on the mechanical properties of wood –polupropylene conposites. Polymers..

[33] López J.P., Gironès J., Mendez J.A., Pèlach M.A., Vilaseca F., Mutjé P. (2013). Impact and flexural properties of stone-ground wood pulp-reinforced polypropylene composites. Polym. Compos..

[34] Herrera-Franco P.J., Valadez-González A. (2005). A study of the mechanical properties of short natural-fiber reinforced composites. Compos. Part B.

[35] Lu J.Z., Wu Q., Negulescu I.I. (2010). Wood-fiber/high-density-polyethylene composites: Coupling agent performance. J. Appl. Polym. Sci..

[36] Gelfuso M.V., Silva P.V.G.D., Thomazini D. (2011). Polypropylene matrix composites reinforced with coconut fibers. Mater. Res..

[37] Azeh Y., Mamza P.A., Olatunji G.A. (2012). Scanning electron microscopy and kinetic studies of ketene-acetylated wood/cellulose high-density polyethylene blends. Int. J. Carbohydr. Chem..

[38] Das S., Saha A.K., Choudhury P.K., Basak R.K., Mitra B.C., Todd T., Lang S., Rowell R.M. (2015). Effect of steam pretreatment of jute fiber on dimensional stability of jute composite. J. Appl. Polym. Sci..

[39] Jiang Z.H. (2013). A comparative in SiO_2_ gel loading performance of wood, bamboo and their carbonized products. Sci. Silvae Sin..

[40] Lee S.Y., Yang H.S., Kim H.J., Jeong C.S., Lim B.S., Lee J.N. (2004). Creep behavior and manufacturing parameters of wood flour filled polypropylene composites. Compos. Struct..

[41] Acha B.A., Reboredo M.M., Marcovich N.E. (2007). Creep and dynamic mechanical behavior of PP–jute composites: Effect of the interfacial adhesion. Compos. Part A.

[42] Bledzki A.K., Faruk O. (2004). Creep and impact properties of wood fibre–polypropylene composites: Influence of temperature and moisture content. Compos. Sci. Technol..

[43] Tamrakar S., Lopez-Anido R.A., Kiziltas A., Gardner D.J. (2011). Time and temperature dependent response of a wood–polypropylene composite. Compos. Part A.

[44] Hristov V., Vasileva S. (2003). Dynamic mechanical and thermal properties of modified poly(propylene) wood fiber composites. Macromol. Mater. Eng..

[45] Mohanty S., Verma S.K., Nayak S.K. (2006). Dynamic mechanical and thermal properties of MAPE treated jute/HDPE composites. Compos. Sci. Technol..

[46] Chang F.C., Kadla J.F., Lam F. (2016). The effects of wood flour content and coupling agent on the dynamic mechanical and relaxation properties of wood-plastic composites. Eur. J. Wood. Wood. Prod..

[47] Nair K.C.M., Thomas S., Groeninckx G. (2001). Thermal and dynamic mechanical analysis of polystyrene composites reinforced with short sisal fibres. Compos. Sci. Technol..

[48] Mohanty S., Nayak S.K. (2006). Interfacial, dynamic mechanical and thermal fiber reinforced behavior of MAPE treated sisal fiber reinforced HDPE composites. J. Appl. Polym. Sci..

[49] López-Manchado M.A., Biagitti J., Kenny J.M. (2002). Comparative study of the effects of different fibers on the processing and properties of ternary composites based on PP-EPDM blends. Polym. Compos..

